# New insights into the immunomodulatory potential of sialic acid on monocyte-derived dendritic cells

**DOI:** 10.1007/s00262-024-03863-7

**Published:** 2024-11-02

**Authors:** Zélia Silva, João Amorim Rabaça, Vanessa Luz, Rita Adubeiro Lourenço, Mariolina Salio, Alexandra Couto Oliveira, Pedro Bule, Sebastian Springer, Paula Alexandra Videira

**Affiliations:** 1 Associate Laboratory i4HB, NOVA School of Science and Technology, Institute for Health and Bioeconomy, Universidade NOVA de Lisboa, 2829-516 Caparica, Portugal; 2 Department of Life Sciences, Applied Molecular Biosciences Unit, UCIBIO, NOVA School of Science and Technology, Universidade NOVA de Lisboa, 2829-516 Caparica, Portugal; 3 Department of Life Sciences, CDG & Allies Professionals and Patient Associations International Network (CDG & Allies–PPAIN), NOVA School of Science and Technology, Universidade NOVA de Lisboa, 2829-516 Caparica, Portugal; 4 Medical Research Council Translational Immune Discovery Unit, Medical Research Council Weatherall Institute of Molecular Medicine, University of Oxford, Oxford, OX39DS UK; 5 CIISA‑Centre for Interdisciplinary Research in Animal Health, Faculty of Veterinary Medicine, University of Lisbon, Avenida da Universidade Técnica, 1300‑477 Lisbon, Portugal; 6 Associate Laboratory for Animal and Veterinary Sciences (AL4AnimalS), 1300‑477 Lisbon, Portugal; 7 Constructor University, 28759 Bremen, Germany

**Keywords:** Immunomodulation, Dendritic cells, Sialic acid, Antigen presentation, MHC-I

## Abstract

Sialic acids at the cell surface of dendritic cells (DCs) play an important immunomodulatory role, and their manipulation enhances DC maturation, leading to heightened T cell activation. Particularly, at the molecular level, the increased stability of surface MHC-I molecules in monocyte-derived DCs (MoDCs) underpins an improved DC: T cell interaction. In this study, we focused on the impact of sialic acid remodelling by treatment with *Clostridium perfringens* sialidase on MoDCs' phenotypic and functional characteristics. Our investigation juxtaposes this novel approach with the conventional cytokine-based maturation regimen commonly employed in clinical settings.

Notably, *C. perfringens* sialidase remarkably increased MHC-I levels compared to other sialidases having different specificities, supporting the idea that higher MHC-I is due to the cleavage of specific sialoglycans on cell surface proteins. Sialidase treatment induced rapid elevated surface expression of MHC-I, MHC-II and CD40 within an hour, a response not fully replicated by 48 h cytokine cocktail treatment. These increases were also observable 48 h post sialidase treatment. While CD86 and PD-L1 showed significant increases after 48 h of cytokine maturation, 48 h post sialidase treatment showed a higher increase in CD86 and shorter increase in PD-L1. CCR-7 expression was significantly increased 48 h after sialidase treatment but not significantly affected by cytokine maturation. Both treatments promoted higher secretion of the IL-12 cytokine. However, the cytokine cocktail induced a more pronounced IL-12 production. SNA lectin staining analysis demonstrated that the sialic acid profile is significantly altered by sialidase treatment, but not by the cytokine cocktail, which causes only slight sialic acid upregulation. Notably, the lipid-presenting molecules CD1a, CD1b and CD1c remained unaffected by sialidase treatment in MoDCs, a finding also further supported by experiments performed on C1R cells. Inhibition of endogenous sialidases Neu1 and Neu3 during MoDC differentiation did not affect surface MHC-I expression and cytokine secretion. Yet, sialidase activity in MoDCs was minimal, suggesting that sialidase inhibition does not significantly alter MHC-I-related functions. Our study highlights the unique maturation profile induced by sialic acid manipulation in MoDCs. These findings provide insights into the potential of sialic acid manipulation as a rapid immunomodulatory strategy, offering promising avenues for targeted interventions in inflammatory contexts.

**Supplementary Information:**

The online version contains supplementary material available at 10.1007/s00262-024-03863-7.

## Introduction

Dendritic cells (DCs) play a pivotal role in orchestrating immune responses, as professional antigen-presenting cells, crucial to activate T cells and generate immunological memory. Due to this role in stimulating the immune response, DCs emerged as a promising resource for immunotherapy, capable of initiating immune response against infected and cancer cells or dampening the immune response in autoimmune diseases and transplantation [1, 2].

The sialic acid content on the cell surface emerges as a finely tuned regulatory mechanism of immune cells, exhibiting variations across cell types, differentiation states and environmental stimuli [3]. In monocyte-derived dendritic cells (MoDCs), the activity of sialyltransferases during differentiation and maturation dynamically modulates the content of cell surface sialylated structures (sialoglycans) [4, 5]. Although much of sialylation regulation has been attributed to sialyltransferases, sialidases also play a role, modulating sialoglycan content. During their differentiation from monocytes, MoDCs overexpress Neu1 and Neu3 [6], which respectively act on glycoproteins and gangliosides. Previous studies show that cell surface sialic acids in DCs contribute to dampen maturation and downregulate the ability to activate T cells [7–11]. This immunomodulation may be operated through the interactions between sialoglycans and sialic acid binding immunoglobulin type lectins (Siglecs), occurring in cis (within the same cell) or in trans (with another cell), triggering inhibitory signalling pathways [12]. Consequently, sialic acid-Siglec engagements are now regarded as novel immune checkpoints, with ongoing development of strategies targeting these axes [13]. These strategies entail using sialidases to remove sialic acids from cancer cells [14, 15], or the inhibition of sialyltransferase activity [16].

Maturation is essential for DCs to effectively respond to pathogens, allowing them to recognize and fight off infection. Microbial sialidases have been found to exert an immunomodulatory effect, particularly in infections, by cleaving sialic acid from the cell surface and escaping protective barriers on host cells [17, 18].

Either in infection or as experimental set up, sialic acid removal from the cell surface of human DCs by sialidases was shown to induce maturation [4, 19], increasing antigen-presenting and co-stimulatory abilities, resulting in higher polarization of T cells towards a T helper type (Th) 1 phenotype. This improves the ability of autologous T cells to mediate tumour cell killing [10]. Additionally, we have demonstrated that the human major histocompatibility complex class I (MHC-I) is one of the sialylated proteins and its function is modulated by the removal of sialic acids, improved cell surface stability in DCs due to reduced turnover rates. This desialylation of DCs resulted in more and earlier immunological synapses with autologous CD8 + T cells, significantly increasing activation of T cells [9]. Additionally, increased general binding avidity between DCs and T cells was observed when a sialic acid-blocking mimetic was used to reduce the sialic acid content of DCs [7], confirming that sialic acid content affects DC: T cell synapse.

DC-based vaccines typically imply isolating DCs’ precursors from the patient’s blood, followed by differentiation, maturation and antigen loading before reintroduction into the patient's body. These vaccines can be tailored to different production methods and therapeutic purposes, namely to promote immunity against cancer or infectious cells or inducing tolerance to specific antigens [20].

In this work, we aimed to further elucidate the effect of sialidase treatment on the maturation features of MoDCs. Our findings reveal that sialic acid manipulation with a sialidase from *Clostridium perfringens* generates MoDCs with a unique maturation profile, not achieved by other sialidases. This profile is characterized by a significant increase in the expression of MHC-I, MHC-II and CD40 immediately after 1 h of sialidase treatment, which extends for 48 h. These effects could not be replicated with a 48 h of conventional cytokine-based maturation cocktail, typically used in clinical settings. A significant increase was also observed for PD-L1, CD86 and CCR-7, 48 h after sialidase treatment. On the other hand, cytokine maturation only affected PD-L1 and CD86 expression, while not being able to significantly increase the expression of CCR-7. IL-12 secretion was significantly increased by both treatments, although maturation with the cytokine cocktail achieved much higher levels. Interestingly, the sialidase treatment improved stabilization of the MHC-I, and MHC-II molecules for up to 48 h, without affecting the levels of other antigen-presentation molecules.

## Materials and methods

### Cells

Human primary DCs were differentiated from monocytes (MoDCs) as previously described [10]. In brief, monocytes were isolated by immunomagnetic separation and cultured in Roswell Park Memorial Institute (RPMI)-1640 medium (ThermoFisher Scientific, cat. no. 1870025), supplemented with 10% foetal bovine serum (FBS, ThermoFisher Scientific, cat. no. 10500064), 2 mM L-glutamine, 100 g/mL penicillin/streptomycin (ThermoFisher Scientific cat. no. 15140122**)**, 1 mM sodium pyruvate (ThermoFisher Scientific, cat. no. 11360070) and 1% non-essential amino acids (ThermoFisher Scientific, cat. no. 11140050), in the presence of 750U/ml of human recombinant interleukin-4 (IL-4) (R&D Systems, cat. no. BT-004) and 1000U/ml of human recombinant granulocyte macrophage colony-stimulating factor (GM-CSF) (Miltenyi Biotec, cat. no. 130–095-372), for 5 to 6 days. At the third day of differentiation, the culture medium was refreshed and supplemented with 750U/ml of IL-4 and 1000U/ml of GM-CSF. The C1R cell lines (human B-cell lymphoblastoid line) transfected with the expression vector containing cDNAs encoding either CD1a, CD1b or CD1c. Cells were cultured in RPMI-1640 medium, supplemented as described above.

### Cell treatments

#### Sialidase

Enzymatic removal of sialic acid from the cell surface was performed using sialidases from *Clostridium perfringens* (Merck/Roche, cat. no. 11585886001), from *Vibrio Cholera* (Merck/Roche, cat. no.7885)*, Macrobdella decora* (Sigma-Aldrich Merck, cat. no. 480706) and O-sialoglycoprotein endopeptidase (Accurate, cat. no. ACLE100) as described in [21]. Briefly, cells (5 × 10^6^ / mL) were resuspended in RPMI medium and 100 mU of sialidase for each 10^6^ cells and incubated for 1 h at 37ºC. After incubation, 1 ml of RPMI medium containing 10% FBS was added to stop the enzymatic reaction, and the cells were centrifuged, discarding the supernatant. To exclude any possible effect not related to sialidase enzymatic activity, a mock-treated control was performed in parallel using the same incubation conditions but with heat-inactivated sialidase.

#### Cytokine cocktail

For the cells treated with the cytokine cocktail, after the differentiation step, the medium was refreshed and a cytokine cocktail comprising interleukin (IL)-1β (Sigma-Aldrich, cat. no. IL038) (10 ng/ml), IL-6 (Merck, cat. no. I1395) (1000 U/ml), prostaglandin E2 (PGE2), (Merck, cat. no. P6532), (1 µg/ml) and TNF-α, (Sigma-Aldrich, cat. no. T6674) (10 ng/ml) was added for 48 h.

#### Sialidase inhibitors

To inhibit endogenous sialidase activity, 1 mM of 2,3-dehydro-2-deoxy-N-acetylneuraminic acid (DANA), (Merck, cat. no. D9050) or 1 mM of 4-guanidino-2,4-dideoxy-2,3-dehydro-N-acetylneuraminic acid (Zanamivir), (Merck, cat. no. SML0492) were added to the culture medium during the differentiation period.

### Flow cytometry analysis

For staining with antibodies and lectins, cells were resuspended (1 × 10^6^ cells/mL) in RPMI supplemented with 10% FBS and stained with the following antibodies or lectins: fluorescein isothiocyanate (FITC)-conjugated anti-MHC-I (W6/32) (Immunotools, cat. no. 21159033); allophycocyanin (APC)-conjugated anti-MHC-II (GRB-1) (Immunostep, cat. no. PHLADRF-100 T); anti-CD1a (OKT6) (Immunotools, cat. no. 21159033); APC-conjugated anti-CD1b (SN13) (Biolegend, cat. no. 32919110); phycoerythrin (PE)-conjugated anti-CD1c (AD5-8E7) (Miltenyi Biotec, cat. no. 1200–000-889); (FITC)-conjugated anti-CD86 (BU63) (Immunotools, cat. no. 21480863); APC-conjugated anti-CD40 (HI40a) (Immunotools, cat. no. 21270406); FITC-conjugated anti-CCR-7 (G043H7) (Biolegend, cat. no. 353216), PE-conjugated anti-PD-L1 (MIH1) (BD Biosciences, cat. no. 557924) and biotin-conjugated *Sambucus nigra* lectin (SNA) (Vector labs, cat. no. B-1305–2). Staining was performed at 4ºC for 30 min in the dark, and cells were washed after. For the SNA staining, streptavidin PE was added and incubated at 4ºC for 30 min in the dark. Quality control of the flow cytometer’s performance and coefficient of variation (CV) values were monitored on a day-to-day basis using performance tracking beads (Thermofisher, cat. no. 4449754).

### Gene expression assay

The RNA was extracted from 1 × 10^6^ MoDCs using the GenElute Mammalian Total RNA Miniprep Kit (Sigma, cat. no. RTN70-1KT). RNA concentration of each sample was determined spectrophotometrically. The RNA was reverse transcribed with random primers using the High-capacity cDNA Reverse Transcription Kit (Thermoscientific/Applied Biosystems, cat. no. 4368814).

Real-time quantitative polymerase chain reaction (RT-qPCR) was performed in Rotor-Gene 6000 Series (Corbett Research Ltd., UK) using TaqMan Universal PCR Master Mix II, TaqMan probes and primers from TaqMan Gene Expression Assays (Thermofisher Scientific). The assay ID provided by the manufacturer are the following: IL-1*β* (Hs00174097_m1), IL-6 (Hs00174131_m1); IL-10 (Hs00174086_m1); IL-12a (Hs00168405_m1); tumour necrosis factor (*TNF-a*; Hs00174128_m1); *glyceraldehyde 3-phosphate dehydrogenase* (*GAPDH*; 4333764F); *β-actin* (4352935E). Each reaction was performed in duplicate. Thermal cycling conditions were 95ºC for 20 s followed by 40–50 cycles of 95ºC for 3 s and 60ºC for 30 s.

Messenger RNA (mRNA) expression was normalized using the geometric mean of the expression of the *GAPDH* and *β-actin* genes as a reference. The relative expression of each gene was calculated according to the 2^−ΔCT^* 1000 method [22]. The efficiency of the amplification reaction for each primer/probe is above 95% (as determined by the manufacturer).

### Cytokine production evaluation

The production of IL-12 was assessed in culture supernatants by an enzyme-linked immunosorbent assay (ELISA) technique using human interleukin-12p40-total development kit (Immunotools, cat. no. 31679129), following the manufacturer’s instructions. Cytokine concentration was calculated using the specific standard curves.

### Measurement of endogenous sialidase activity.

Sialidase activity was measured in lysates from MoDCs (0.5 × 10^6^ cells) that were not stimulated or stimulated with 5 or 50 µg/mL of lipopolysaccharide (LPS) for 60 min. The assay mixture consisted of 200 mM citrate–phosphate buffer (pH 4.5) containing 0.1% BSA (w/v), 0.5 mM4MU-Neu5Ac (TCI Chemicals) and the sample fractions (10 µL) in a final volume of 100 µL. After incubation for 30 min at room temperature, the reaction was terminated by the addition of 100 µL of 1 M Na_2_CO_3_ (pH 10.4), and the amount of 4MU released was determined fluorometrically with a FLUOstar Optima microplate reader (BMG Labtech) using an excitation wavelength of 360 nm and emission wavelength of 450 nm. One unit of sialidase activity was defined as the amount of enzyme that released 1 µmol sialic acid/min at 37ºC.

### Confocal laser scanning microscopy

To perform confocal laser scanning microscopy, cells were plated on 12-mm diameter polylysine-coated glass coverslips and incubated for 5 min at room temperature. Coverslips were then centrifuged at 100 × g for 1 min to promote cell adhesion, fixed for 30 min with 4% paraformaldehyde (PFA) and washed using 1% bovine serum albumin (BSA) in PBS. Mouse anti-human HLA-ABC, clone 246-B8.E7 (Invitrogen. cat. no. 11504371) was used for staining human MHC-I, followed by a fluorescently conjugated secondary antibody. Images were acquired on a Zeiss LSM710 confocal microscope (Zeiss). Illustrative confocal cross-sectional pictures were selected after Z-stacking processing. Staining intensity was analytically quantified using the corrected total cell fluorescence (CTCF) = Integrated Density—(Area of selected cell × Mean fluorescence of background readings).

### Statistical analysis

Statistical analysis was performed using GraphPad Prism 8.0 software (GraphPad Software, USA). Unless otherwise stated, statistical significance (*p*-value) was calculated using the ratio-paired t-test. Statistical significance was defined as *p* < 0.05 (*), *p* < 0.01 (**) and *p* < 0.001 (***).

### Data availability

Data were generated by authors and are available on request.

## Results

### The modulation of MHC-I expression by sialic acid removal in MoDCs is dependent on the nature of the sialidase

In our previous study, we demonstrated that treatment of immature MoDCs with a sialidase from *C. perfringens* led to an increase in the cell surface expression of MHC-I, attributed to enhanced stability of MHC-I molecules at the cell surface [9]. To assess which sialoglycans influenced MHC-I stability, we used sialidases from various sources as described in the Material and Methods section. These sialidases modulate the cell surface sialome with different specificities: sialidase from *C. perfringens* cleaves α2,3, α2,6 and α2,8-linked sialic acids; sialidase from *V. cholerae* removes α2,3-linked sialic acids decorating gangliosides (sialic acid-containing lipids); sialidase from *M. decora* acts on sialic acid α2,3-linked to galactose; and O-sialoglycoprotein endopeptidase specifically cleaves O-sialoglycoproteins. The activities of these enzymes towards sialoglycans in MoDC's surface was confirmed by staining with PNA lectin and evaluation by flow cytometry **(**Supplementary Figure S1**).**

**Fig. 1:**
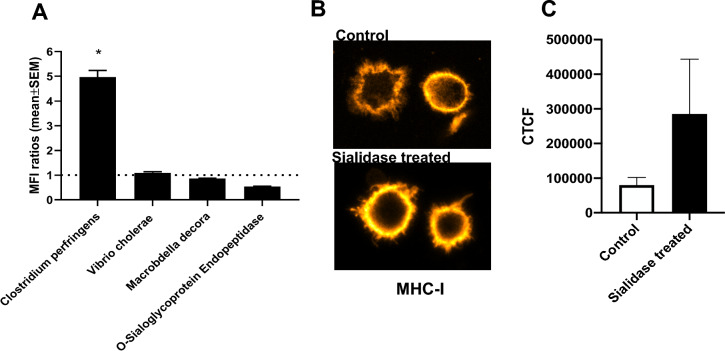
Expression of MHC-I in MoDCs surface is affected by sialidase treatment, and the effect is dependent on the origin of the sialidases**.** Assessment of MHC-I expression after treatment of MoDCs with sialidases **A** MHC-I expression levels were assessed by flow cytometry after MoDCs were treated with different sialidases. Bars represent MFI ratios (mean ± SEM) of the MFI of sialidase-treated cells in relation to the MFI of untreated cells from at least three different donors. Statistically significant differences are indicated by asterisks (* *p* < 0.05). **B** Confocal microscopy images of staining of MHC-I surface expression in MoDCs treated with *C. perfringens* sialidase (bottom image) or left untreated (upper image). **C** MHC-I surface expression levels were assessed by Confocal microscopy in MoDCs treated with *C. perfringens* sialidase (sialidase treated) or left untreated (Control). The fluorescence of MHC-I obtained by confocal microscopy was quantified through the corrected total cell fluorescence (CTCF) method as described in the Methods section. Values presented are mean ± SEM (n ≥ 4)

Treatment with the sialidase from *C. perfringens* resulted in a remarkable fivefold increase in MHC-I levels compared to untreated cells, whereas other sialidases showed no significant effect **(**Fig. 1A**)**. This increase in the expression of MHC-I at the cell surface was confirmed by confocal microscopy images showing higher fluorescence intensity for MHC-I staining in *C. perfringens* sialidase-treated MoDCs, compared to untreated cells (Figs. 1B-C). Notably, *C. perfringens* sialidase treatment also augmented MHC-I expression in THP-1 cells, another antigen-presenting monocytic cell model (**Supplementary Figure S2**), suggesting that the observed mechanism is shared by different antigen-presenting cells. These findings suggest that the modulation of MHC-I expression in MoDCs is intricately linked to the trimming of *α*2,3, *α*2,6-linked sialic acids, mostly from proteins. *C. perfringens* sialidase was hereby used to manipulate the sialic acid content of MoDCs in the subsequent assays.

### 3.2 Sialidase-treated MoDCs show differential phenotypic and functional characteristics when compared to a cytokine maturation cocktail used in clinical trials.

**Fig. 2:**
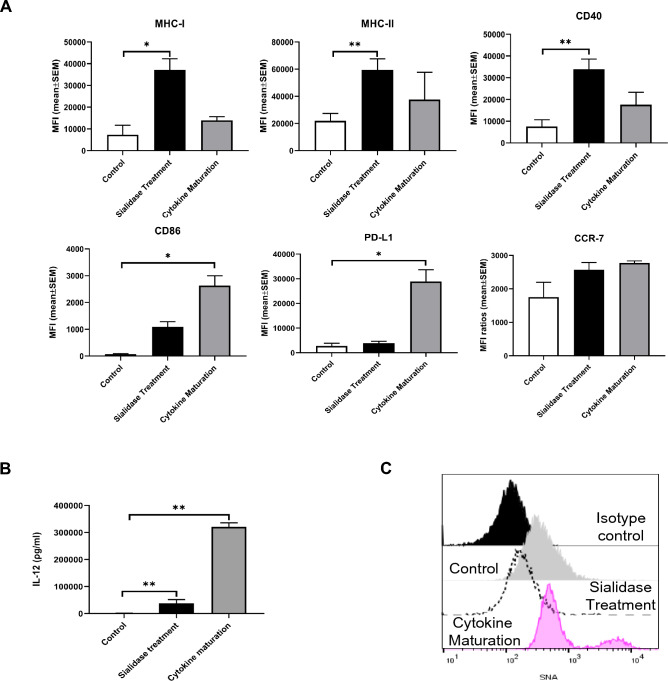
Sialidase-treated MoDCs show differential phenotypic and functional characteristics, when compared to MoDCs treated with a cytokine maturation cocktail. Evaluation of maturation profile of MoDCs submitted in parallel to sialidase treatment for 1 h and to cytokine maturation cocktail for 48 h **A**. Maturation marker's expression levels were assessed by flow cytometry. Bars represent MFI (mean ± SEM) from MoDCs obtained, at least, from three different donors, **B** IL-12 secretion was measured by ELISA in culture supernatants of cells cultured for 48 h after treatments. Cytokine maturation represents MoDCs differentiated from monocytes and then maturated for 48 h with a cytokine cocktail composed of IL-1β (10 ng/mL), IL-6 (1000 U/mL), TNF-α (10 ng/mL) and PGE2 (1 µg/mL). **C** Evaluation of sialic acid content of MoDCs subjected to different treatments. Sialic acid content was evaluated by SNA staining. Representative histograms of sialidase-treated MoDCs versus MoDCs treated with cytokine maturation. Statistical significance analysis was performed using ratio-paired t-test. *p* < 0.05 (*), *p* < 0.01 (**)

We have previously shown that the removal of sialic acids by sialidase treatment induces DC maturation within an hour, as evidenced by increased expression of antigen-presenting and co-stimulatory molecules, suggesting a potential new technology to mature DC to be used in clinical settings [9]. Moreover, the ability of dendritic cells treated with sialidase to improve T cell proliferation was proved in a mixed lymphocyte reaction (**Supplementary Figure S3**). To better understand the clinical potential of the sialidase effect, we compared its effect with the maturation protocol used in clinics, namely the “gold standard” maturation cocktail composed of IL-1β, IL-6, PGE2 and TNF-α for 48 h [23]. As shown in Fig. 2, both treatments increased the surface expression of MHC molecules and maturation markers. Immediately after sialidase treatment, there was a significant fivefold, threefold and fivefold increase of MHC-I, MHC-II and CD40, respectively, while cytokine cocktail led to a non-significant twofold increase for the same molecules. On the other hand, with cytokine cocktail, there was a significant 37-fold and 11-fold increase in CD86 and PD-L1, respectively, whereas immediately after sialidase treatment, there was only 15-fold and twofold marginal increase in CD86 and PD-L1, respectively. For CCR-7, there was a negligible 1.6-fold increase with the cytokine cocktail and 1.5-fold increase immediately after sialidase treatment **(**Fig. 2A**)**. To assess the effect of sialidase treatment over the same time as the cytokine cocktail, we analyse its impact after 48 h post sialidase treatment, CD86, PD-L1 and CCR-7 levels significantly increased by 167-fold, fourfold and 2.6-fold, respectively (Table 1**)**.

**Table 1:** Cell surface expression of maturation markers in MoDCs after sialidase treatment (0 h and 48 h) or cytokine maturation (48 h)

	Control	Sialidase treatment				Cytokine maturation	
	0 h^#^	0 h^#^	48 h^#^			48 h^#^	
	MFI (mean ± SEM)	MFI (mean ± SEM)	*P*	MFI (mean ± SEM)	*P*	MFI (mean ± SEM	*P*
CD86	71 ± 26	1094 ± 191	ns	11,879 ± 750	*	2632 ± 368	*
PD-L1	2718 ± 1153	3954 ± 737	ns	12,080 ± 3892	***	28,870 ± 4828	*
CCR-7	1757 ± 441	2570 ± 217	ns	4588 ± 110	*	2774 ± 59	ns
MHC-I	7312 ± 4370	37,141 ± 5172	*	51,769 ± 8430	*	13,961 ± 1700	ns
MHC-II	22,043 ± 5377	59,476 ± 8158	*	450,290 ± 79,416	*	37,681 ± 20,079	ns
CD40	7574 ± 3064	33,978 ± 4643	*	420,429 ±85,180	*	17,611 ± 5741	ns

ns-not significant, # time post-treatment. Cytokine maturation represents MoDCs differentiated from monocytes and then maturated for 48 h with a cytokine cocktail composed of IL-1β (10 ng/mL), IL-6 (1000 U/mL), TNF-α (10 ng/mL) and PGE2 (1 µg/mL). Statistical significance was performed against the control, using ratio-paired t-test, *p* < 0.05 (*), *p* < 0.01 (**) and *p* < 0.001 (***)

The Th1-inducing cytokine, IL-12 was measured in the culture supernatants after 48 h of cell culture post-with either sialidase or with the cytokine cocktail. The levels of IL-12 increased approximately 26 times with the sialidase treatment and 223 times with the cytokine cocktail **(**Fig. 2B**)**. To evaluate sialic acid content after sialidase and cytokine cocktail treatments, MoDCs were stained with the SNA lectin and evaluated by flow cytometry. While, as expected, sialidase treatment decreased the staining with SNA resulting from the removal of α-2,6 sialic acids, cytokine maturation caused a non-significant increase in SNA staining **(**Fig. 2C**)**.

To understand whether sialidase treatment induces significant transcriptional changes of cytokines, we evaluated the expression of genes (IL-12, TNF-α, IL-1β, IL-10 and IL-6) by RT-PCR. At 3 h, the sialidase-treated MoDCs have a more pronounced transcription of IL-12 than MoDCs treated with a cytokine cocktail (**Supplementary S4**). After 6 h, the transcriptional levels were lowered for sialidase-treated cells and were highly increased for MoDCs treated with the maturation cocktail. For TNF-α, the mRNA levels are higher at 3 h and 6 h for MoDCs treated with the cytokines, while for IL-1β, the mRNA levels are higher for sialidase-treated MoDCs than for the cytokine maturated MoDCs. For IL-10 and IL-6, the mRNA levels are very low for all the tested conditions (**Supplementary Figure S4**). These cytokine expression profiles highlight critical differences between sialidase and cytokine stimulation, with potential clinical interest. Altogether, these data reinforce the potential clinical benefit of the sialidase effect, as a rapid strategy to improve peptide presentation and CD40 co-stimulation and a better ability to control PD-L1 expression in MoDC maturation.

### Sialidase treatment differently affects the expression of CD1 lipid-presenting molecules

**Fig. 3:**
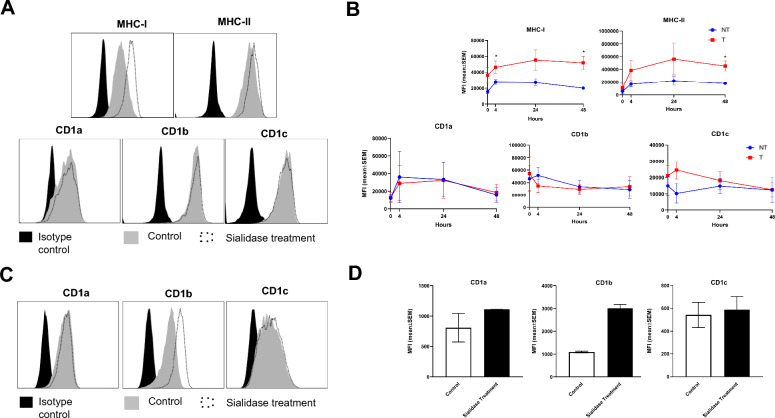
Sialidase treatment affects differently the expression of CD1 molecules at the cell surface. **A** Representative histograms from the staining of MoDCs with mab against different antigen-presenting molecules immediately after sialidase treatment for 1 h as described in Materials and Methods. **B** After sialidase treatment for 1 h the dendritic cells were immediately analysed (0 h) or were put back in culture for 4, 24 and 48 h. After each time point, the cells were collected and the expression of molecules at cell surface was evaluated by flow cytometry. **C** Representative histograms from the staining of C1R cells with mab against CD1a, CD1b and CD1c lipid antigen-presenting molecules, immediately after sialidase treatment for 1 h**.** **D** The expression of CD1a, CD1b and CD1c lipid-presenting molecules at C1R cell surface as assessed by flow cytometry, immediately after sialidase treatment for 1 h. Bars represent MFI (mean ± SEM) from at least three different donors. Statistical significance was performed using ratio paired t-test. *p* < 0.05 (*), *p* < 0.01 (**) and *p* < 0.001 (***)

We then investigated the impact of sialidase treatment on the expression of CD1 lipid-presenting molecules, to determine whether it parallels the observed enhancement in MHC class I molecules. CD1a, CD1b and CD1c share sequence and structural homology with MHC class I, and are also decorated with sialic acid-containing glycans [9]. To assess the impact of sialic acid removal on the levels of CD1 molecules over time, the DCs were evaluated immediately after sialidase treatment and at subsequent time points during culture **(**Figs. 3A, B**)**. As expected, the augmented MHC-I and MHC-II expression levels of sialidase-treated cells was kept over time **(**Fig. 3B**)**. However, for the lipid-presenting molecules CD1a, CD1b and CD1c there were no significant differences between the non-treated and treated cells at any of the points tested (Figs. 3A, B). Additionally, we tested the effect of sialidase treatment on the human B-cell lymphoblastoid line C1R, transfected with CD1a, CD1b or CD1c. While the levels of CD1a, CD1c were not affected by sialidase treatment, a negligible impact was observed on CD1b levels (Figs. 3C, D). From these observations, we can conclude that sialic acid removal in MoDCs improves the expression of MHC but not CD1 molecules, suggesting that it affects only peptide but not lipid antigen presentation.

### Inhibition of endogenous sialidases during monocyte differentiation does not affect MoDCs

**Fig. 4:**
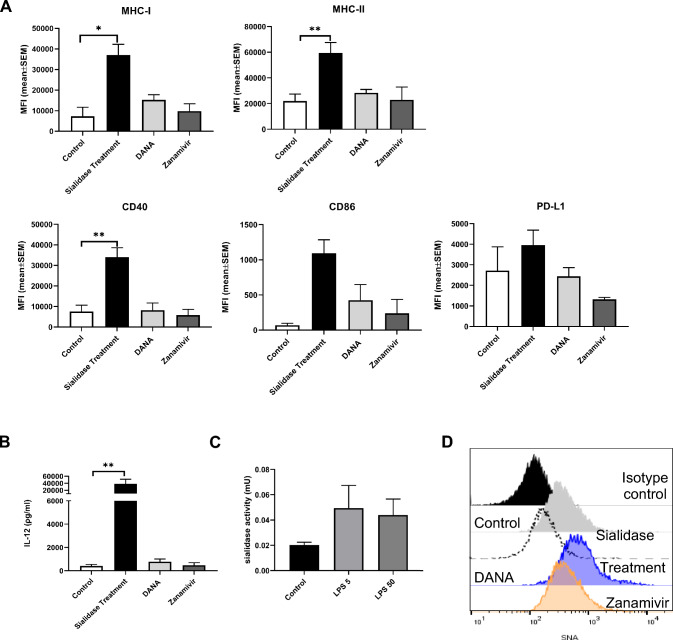
Inhibition of endogenous sialidases does not alter MoDC’s phenotype. Evaluation of maturation profile of dendritic cells differentiated in the presence of inhibitors of endogenous sialidases (DANA or Zanamivir) **A** Maturation markers expression levels were assessed by flow cytometry. Bars represent MFI (mean ± SEM) from at least three different donors **B** IL-12 secretion was measured by ELISA in culture supernatants after differentiation without endogenous sialidase inhibitors (control) and in the presence of DANA or Zanamivir and after sialidase treatments. **C** Evaluation of sialidase activity in lysates of immature dendritic cells (control) or after stimulation with 5 or 50 µg/mL of LPS for 60 min. The endogenous sialidase activity was evaluated, using M4MU-Neu5Ac as the substrate. One unit of sialidase activity was defined as the amount of enzyme that released 1 µmole sialic acid/h at 37 °C. **D** Evaluation of sialic acid content of MoDCs subjected to different treatments. Sialic acid content was evaluated by SNA staining. Representative histograms of sialidase-treated MoDCs versus MoDCs treated with DANA and Zanamivir. Statistical significance was performed using ratio-paired t-test. *p* < 0.05 (*) and *p* < 0.01 (**)

MoDCs express the endogenous sialidases Neu 1 and Neu 3, which act preferentially on glycoproteins and gangliosides, respectively [6]. To assess the potential impact on MoDCs’ phenotype of inhibiting Neu 1 and Neu 3 activity during their differentiation from monocytes, we used two inhibitors: DANA, a potent Neu1 and Neu3 inhibitor, or Zanamivir, a strong Neu3 inhibitor. Monocytes were differentiated in the presence of these inhibitors, and the expression of cell surface markers (MHC-I, MHC-II, CD40, CD86 and PD-L1) was assessed by flow cytometry. Interestingly, the inhibition of endogenous sialidases by DANA or Zanamivir did not alter the expression of the DC surface markers **(**Fig. 4A**)**. Accordingly, the levels of the cytokine IL-12, measured in the culture supernatants, showed no difference for the DCs differentiated in the presence of the inhibitors when compared to DCs cultured with the control differentiation medium **(**Fig. 4B**).** To evaluate MoDCs endogenous sialidase activity 4MU-Neu5Ac was used as the substrate. The activity detected for immature MoDCs (control) was 0.020 ± 0.002 mU and for MoDCs stimulated for 60 min with LPS 5 and 50 µg/mL was 0.049 ± 0.018 mU and 0.044 ± 0.013 mU, respectively (Fig. 4C). Interestingly, when sialidase inhibitors were added to the differentiation medium, the staining of MoDCs with SNA showed no significant differences. However, in comparison to the control, there was a slight increase in α-2,6-linked sialic acids, especially in the presence of DANA, the inhibitor of Neu1 and Neu3 **(**Fig. 4D). While the staining results hint at subtle alterations in α-2,6-linked sialic acids, the overall impact of sialidase inhibitors appears negligible. In conclusion, in these experimental conditions, MoDCs showed very low sialidase activity, supporting the observation that the use of sialidase inhibitors does not significantly affect their phenotype.

## Discussion

The field of DC-based immunotherapy has witnessed a very promising but also challenging development with clinical trials evolving slowly due to concerns about effectiveness. Innovative approaches which improve DC-based vaccines are needed to increase their efficacy [24]. Consequently, there is a pressing need to identify and understand mechanisms and complementary forms of maturing DCs.

While different methods exist for DC production, particular emphasis is placed on refining the methodology for generating DC derived from monocytes (MoDCs) due to their clinical potential. This method benefits from the existence of reliable techniques for large-scale production and widespread application in clinical settings [25, 26].

Since DC's ability to present antigens is dependent on the MHC-I, understanding the dynamic of MHC-I, including its recycling and stabilization mechanisms in DCs is critical to improve effective clinical applications.

DCs exhibit a cell surface adorned with sialic acid, tightly regulated, and dynamically altered during differentiation and stimulation. We have previously shown that enforced desialylation improves expression of MHC-I at DC’s surface. When removing the sialic acid from the surface many glycoproteins and gangliosides may be affected. Sialic acid trimming using sialidases with different specificities target different molecules. Since MHC-I molecules was only affected by sialidase from *C. perfringens* which cleaves α2,3, α2,6 and α2,8-linked sialic acids and not by other sialidases points to an effect prominently dependent on N-glycosylation instead of O-sialoglycoproteins (O-sialoglycoprotein endopeptidase), on proteins instead of gangliosides (*V. cholerae)* and mainly dependent on α2,6 and not α2,3-linked to galactose (*M. decora)*.

Interestingly, here, through our experiments with different sialidases, we have shown that the modulation of MHC-I expression in MoDCs is linked to the trimming of α2,6-linked sialic acid by *C. perfringens* sialidase, which also trims α2,3 and α2,8-linked sialic acids from cell glycoproteins. To exclude any concern on the possible contamination of sialidases with endotoxins in the purified enzymes, we developed preliminary assays with heat-inactivated sialidases. When the dendritic cells were treated with heat-inactivated sialidases, we observed no effect on the maturation. This observation excludes, therefore, the possibility that the maturation is caused by the presence of endotoxin contamination in the purified enzymes. Furthermore, there are other pieces of evidence that sialic acid shortage also leads to maturation without using sialidase. As example, ST6Gal1 deficient mice also produced dendritic cells with a more mature phenotype [27]. Our results corroborate previous studies in leukaemia cells comparing sialidase effects, including *C. perfringens*, and demonstrated enhanced stimulatory capacity linked to the removal of specific membrane structures, likely α2-6 linked to N-linked glycosyl moieties [28].

These findings add further insights into the already-known role of N-glycans in the dynamics of MHC-I [29], its conformation and strength of the TCR-MHC-I complex [30, 31], highlighting the role of α2-6 sialylated N-glycans. These results extend to the recently revealed sialic acid impact on neoglycosphingolipids, indirectly influencing the function of MHC-I, by shielding and hindering its recognition [32].

Our findings uniquely position the role of sialic acids as immune regulators, besides engaging with Siglecs on immune cells, and serving as immune checkpoints, akin to PD-1 and CTLA-4 receptors [33]. Furthermore, our findings underscore the relevance of sialidase technology as a tool for manipulating sialic acid content, thereby influencing the immune response.

Structurally similar to Siglecs, the T cells co-stimulation receptor CD28 also engages with sialic acids, either in cis or trans. This interaction with sialic acids competes with binding to the activator ligand CD80 on antigen-presenting cells, leading to attenuation of T cell responses [11]. Although there are reports on the immunoregulatory role of sialic acids in DCs [34], we were the first to report their influence in MHC-I, leading to reduced internalization and higher stability accounting for the increased expression on the cell surface.

Interestingly, others demonstrated that depleting sialic acid in cancer cells disrupts intracellular trafficking, resulting in diminished recycling of internalized receptors and enhanced endosomal delivery [35]. Therefore, it is likely that besides MHC-I, other cell surface molecules also experience delayed trafficking in desialylated cells. Although to depict the specific effect of sialic acid on the stability and/or turnover for the other molecules a full mechanistic characterization, similar to the performed for MHC-I, would be required. While our work did not investigate its turnover, the fact that increased expression in some of the molecules after sialidase treatment was immediately observed after 1 h, it is unlikely that they represent changes on protein expression that would require several hours to occur. For that reason, RNA transcription was not evaluated.

Surprisingly, the sialidase treatment did not affect the levels of the MHC-I-like glycoproteins-CD1 molecules, pointing to distinctive features of these molecules or neighbouring molecules at the cell surface that make them unaffected by the action of sialidase. CD1 molecules present lipid antigens and, in DCs, do not undergo the same changes in intracellular trafficking as MHC-I during DC maturation [36, 37].

The role of glycosylation of MHC-I in assembling, epitope selection and antigen presentation, which occurs through chaperones and modifying enzymes, is well documented [38]. The assembly of MHC-I is dependent on calreticulin binding to the monoglycosylated branch of the N-glycan, and its interaction with tapasin is also glycan dependent involving ERp57 and calreticulin [38]. Importantly, the sialic acid removal using sialidase, described in this work, is unlikely to affect these processes because it is restricted to the cell surface when the MHC-I complex is already formed, being advantageous over other more drastic sialic acid removal strategies. Sialic acid is a charged monosaccharide capping glycan chains, whose role in protein turnover has been debated since desialylation does not affect the internalization of several cell surface glycoproteins [39]. Yet, these studies have not enclosed MHC-I in human DCs, which, collectively with our observation that other molecules are not affected by sialidase, suggest that sialic acid affects the turnover of only specific proteins, potentially present in specific cell contexts. Moreover, the increase in MHC-I onTHP-1 cell line corroborate the results obtained for MoDCs, suggesting that the observed mechanism is shared by different antigen-presenting cells. Yet, for cancer cell lines, the surface content of MHC-I was unaffected by desialylation, pointing to a restricted action towards immune APCs [9].

Comparative analyses between sialidase treatment and cytokine maturation underscored distinct phenotypic and functional characteristics in MoDCs. DC vaccine's *ex vivo* production aims for the expression of co-stimulatory molecules, low expression of immune inhibitory molecules, capacity for T cell polarization towards Th1 characterized by their ability to produce IL-12 upon contact with responding T cells, expression of CCR-7 to improve migration to lymph nodes and superior capacity to activate cytotoxic T cell responses [40]. By comparing the effects of sialidase treatment with cytokine maturation, we essentially observe differences on the levels and the rate of expression of surface molecules. Sialidase treatment produced a very rapid (immediately after 1 h) and steady increase of MHC-I, and other molecules for at least 48 h, without significantly increasing PD-L1. Strategies to upregulate MHC-I without increasing the PD-L1 are very important. Therefore, sialidase treatment may be a very efficient strategy to rapidly improve antigen presentation through MHC-I as well as other critical molecules without increasing inhibitory effects. Another very important molecule that was significantly upregulated only by sialidase treatment and not by cytokine cocktail was CCR-7, which enhances DC migration to lymph nodes to encounter T cells.

Furthermore, the production of cytokines by DCs during the maturation process determines the immune responses generated by CD4^+^ T cells [41]. Both sialidase treatment and maturation with cytokines upregulated the secretion of the Th1-inducing cytokine IL-12 at transcriptional and protein level. IL-12 is critical for the proliferation and lytic activity on T cells, stimulating the secretion of IFN and anti-tumour function [40, 42]. Besides IL-12, the expression of cytokines such as TNF-α, IL1β and IL-6 is also critical for the activation of anti-tumour immune responses [43]. Importantly, the treatment with sialidase significantly increased the transcription of IL-1β and IL-6 genes which are inhibited by the cytokine cocktail. The signalling induced by these cytokines activates immune cells and drives polarization of CD4 + T cells towards Th1 and Th17 cells with a largely beneficial role in initiating adaptive anti-tumour responses [44]. It is likely that the cytokine cocktail, containing IL-1β and IL-6, inhibits the expression of those cytokines by DCs by negative feedback, in agreement with the known IL-1β and IL-6 interplay [45]. In contrast, sialidase treatment overcomes negative feedback, and the NF-κB pathway required for IL-6 and IL-1β expression is activated upon sialidase treatment of MoDCs [19]. Thus, sialidase treatment emerges as a reliable alternative strategy to induce DC maturation and sustain appropriate cytokine intracellular signalling.

In MoDC, endogenous sialidase activity has been associated with LPS-induced cytokine production^6^. In our study, the expression of MoDCs surface markers was unchanged after treatment with inhibitors DANA and Zanamivir. Regarding the effects of these inhibitors, although it was reported that the LPS-induced cytokine production by MoDCs was affected by them, the expression of surface markers was unchanged,6which is in agreement with our results. Moreover, in our experimental conditions, we observed very little sialidase activity in control conditions and that activity was only slightly increased by LPS, which may explain the null effect of sialidase inhibitors on the phenotype of MoDCs. From these observations, we conclude that endogenous sialidase inhibition cannot be considered as a strategy to increase sialic acid content in MoDCs. However, it is possible to modulate the sialic acid content in MoDCs ex vivo either by removing sialic acids using sialidase as shown in this work or by increasing its content by employing sialyltransferases externally, producing a destabilizing effect on MHC-I, opposite to stabilizing effect produced by sialic acid removal [9].

Finding the optimized maturation protocol has been a challenge not yet surpassed due to the difficulty to replicate the natural body environment *ex vivo*, which endows DCs with ideal antigen-presentation, co-stimulatory abilities and also migratory capacity towards the lymph nodes [46]. In this work, we present new insights on the potential of the manipulation of sialic acid content as a means to tailor the production of DCs for clinical applications.

## Supplementary Information

Below is the link to the electronic supplementary material.

Supplementary file1 (PPTX 897 KB)

## Data Availability

No datasets were generated or analysed during the current study.
