# Supplementary material for: New insights into the immunomodulatory potential of sialic acid on monocyte-derived dendritic cells
Source: Cancer Immunol Immunother. 2024 Nov 2;74(1):9. doi: 10.1007/s00262-024-03863-7 (PMC11531459; doi:10.1007/s00262-024-03863-7)
Supplement: Supplementary file 1 — Supplementary file1 (PPTX 897 KB) [file 262_2024_3863_MOESM1_ESM.pptx]

## Slide 1
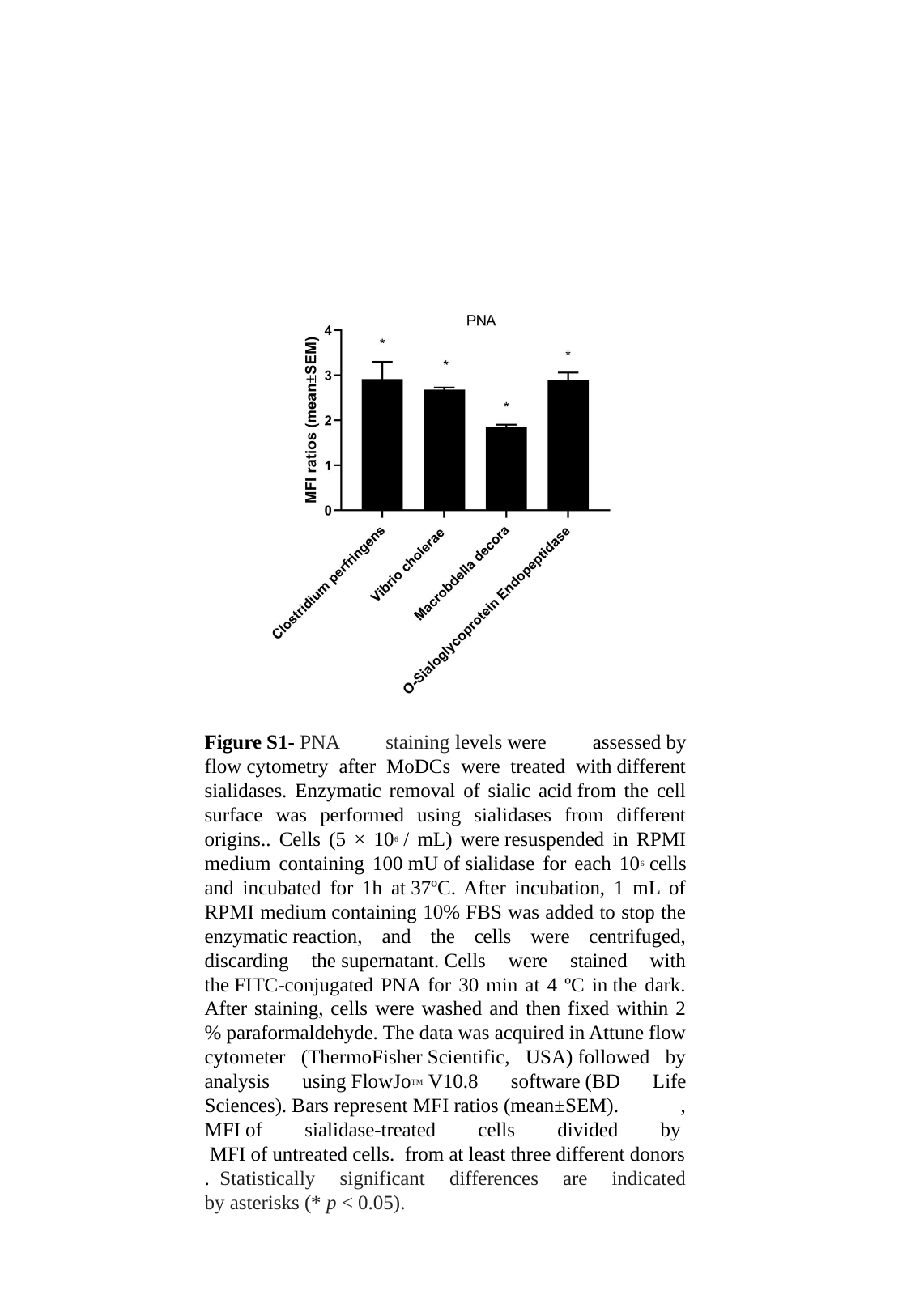

Figure S1- PNA staining levels were assessed by flow cytometry after MoDCs were treated with different sialidases. Enzymatic removal of sialic acid from the cell surface was performed using sialidases from different origins.. Cells (5 × 10^6^ / mL) were resuspended in RPMI medium containing 100 mU of sialidase for each 106 cells and incubated for 1h at 37ºC. After incubation, 1 mL of RPMI medium containing 10% FBS was added to stop the enzymatic reaction, and the cells were centrifuged, discarding the supernatant. Cells were stained with the FITC-conjugated PNA for 30 min at 4 ºC in the dark. After staining, cells were washed and then fixed within 2 % paraformaldehyde. The data was acquired in Attune flow cytometer (ThermoFisher Scientific, USA) followed by analysis using FlowJoTM V10.8 software (BD Life Sciences). Bars represent MFI ratios (mean±SEM). , MFI of sialidase-treated cells divided by   MFI of untreated cells.  from at least three different donors.  Statistically significant differences are indicated by asterisks (* p < 0.05).

## Slide 2
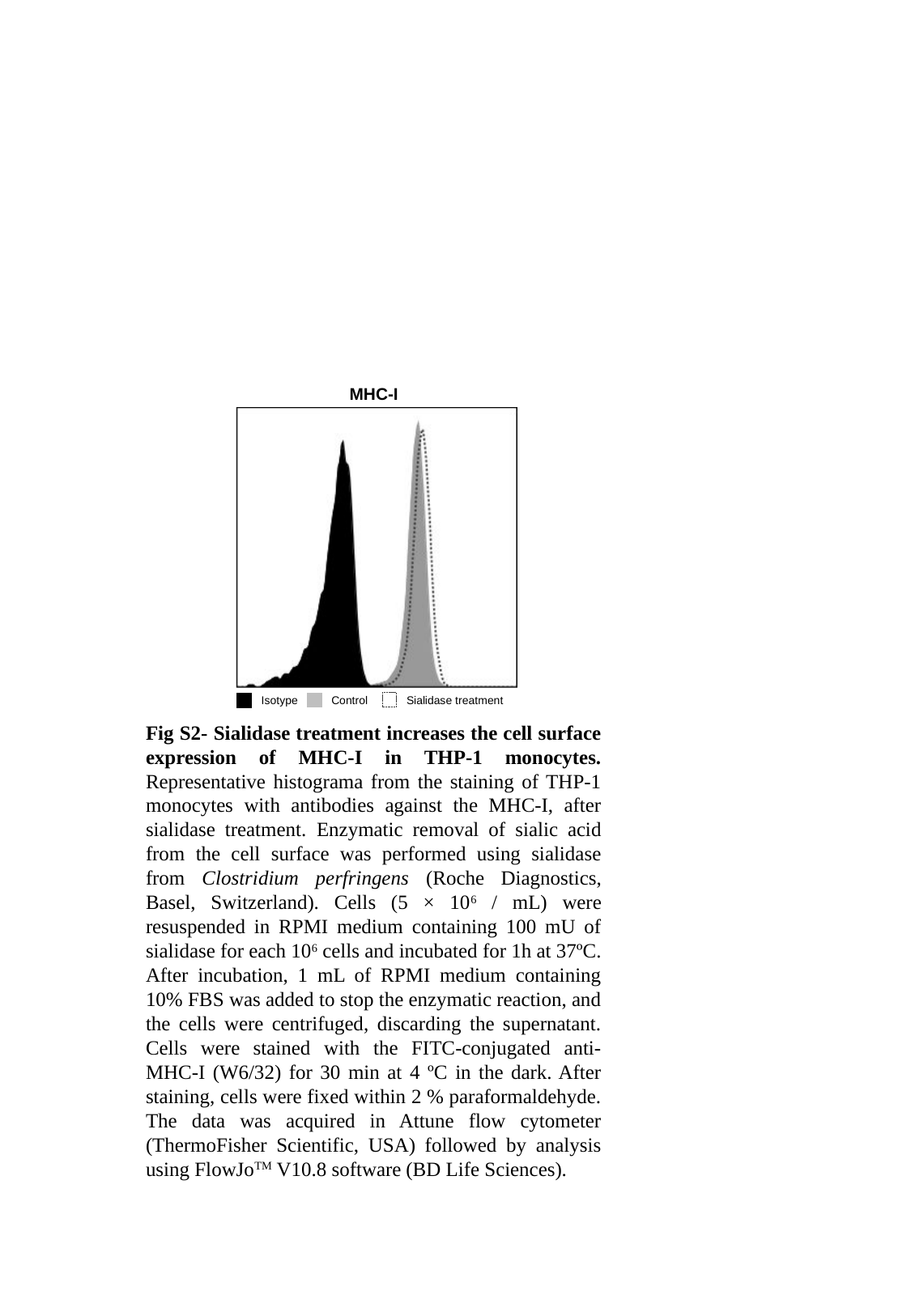

MHC-I
Isotype
Sialidase treatment
Control
Fig S2- Sialidase treatment increases the cell surface expression of MHC-I in THP-1 monocytes. Representative histograma from the staining of THP-1 monocytes with antibodies against the MHC-I, after sialidase treatment. Enzymatic removal of sialic acid from the cell surface was performed using sialidase from Clostridium perfringens (Roche Diagnostics, Basel, Switzerland). Cells (5 × 10^6^ / mL) were resuspended in RPMI medium containing 100 mU of sialidase for each 106 cells and incubated for 1h at 37ºC. After incubation, 1 mL of RPMI medium containing 10% FBS was added to stop the enzymatic reaction, and the cells were centrifuged, discarding the supernatant. Cells were stained with the FITC-conjugated anti-MHC-I (W6/32) for 30 min at 4 ºC in the dark. After staining, cells were fixed within 2 % paraformaldehyde. The data was acquired in Attune flow cytometer (ThermoFisher Scientific, USA) followed by analysis using FlowJoTM V10.8 software (BD Life Sciences).

## Slide 3
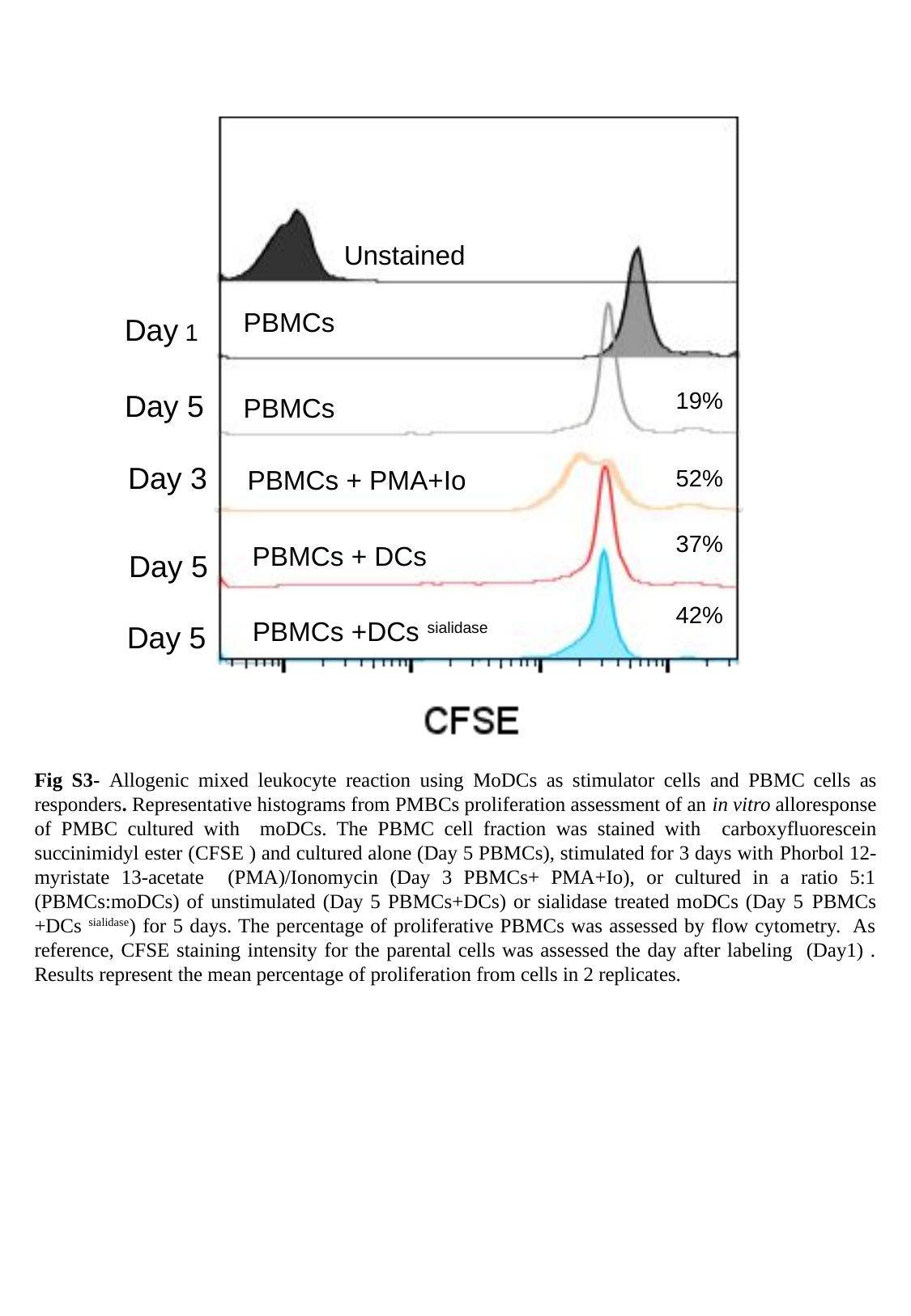

Unstained
PBMCs
Day 1
19%
Day 5
PBMCs
Day 3
52%
 PBMCs + PMA+Io
37%
 PBMCs + DCs
Day 5
42%
PBMCs +DCs sialidase
Day 5
Fig S3- Allogenic mixed leukocyte reaction using MoDCs as stimulator cells and PBMC cells as responders. Representative histograms from PMBCs proliferation assessment of an in vitro alloresponse of PMBC cultured with moDCs. The PBMC cell fraction was stained with carboxyfluorescein succinimidyl ester (CFSE ) and cultured alone (Day 5 PBMCs), stimulated for 3 days with Phorbol 12-myristate 13-acetate (PMA)/Ionomycin (Day 3 PBMCs+ PMA+Io), or cultured in a ratio 5:1 (PBMCs:moDCs) of unstimulated (Day 5 PBMCs+DCs) or sialidase treated moDCs (Day 5 PBMCs +DCs sialidase) for 5 days. The percentage of proliferative PBMCs was assessed by flow cytometry. As reference, CFSE staining intensity for the parental cells was assessed the day after labeling (Day1) . Results represent the mean percentage of proliferation from cells in 2 replicates.

## Slide 4
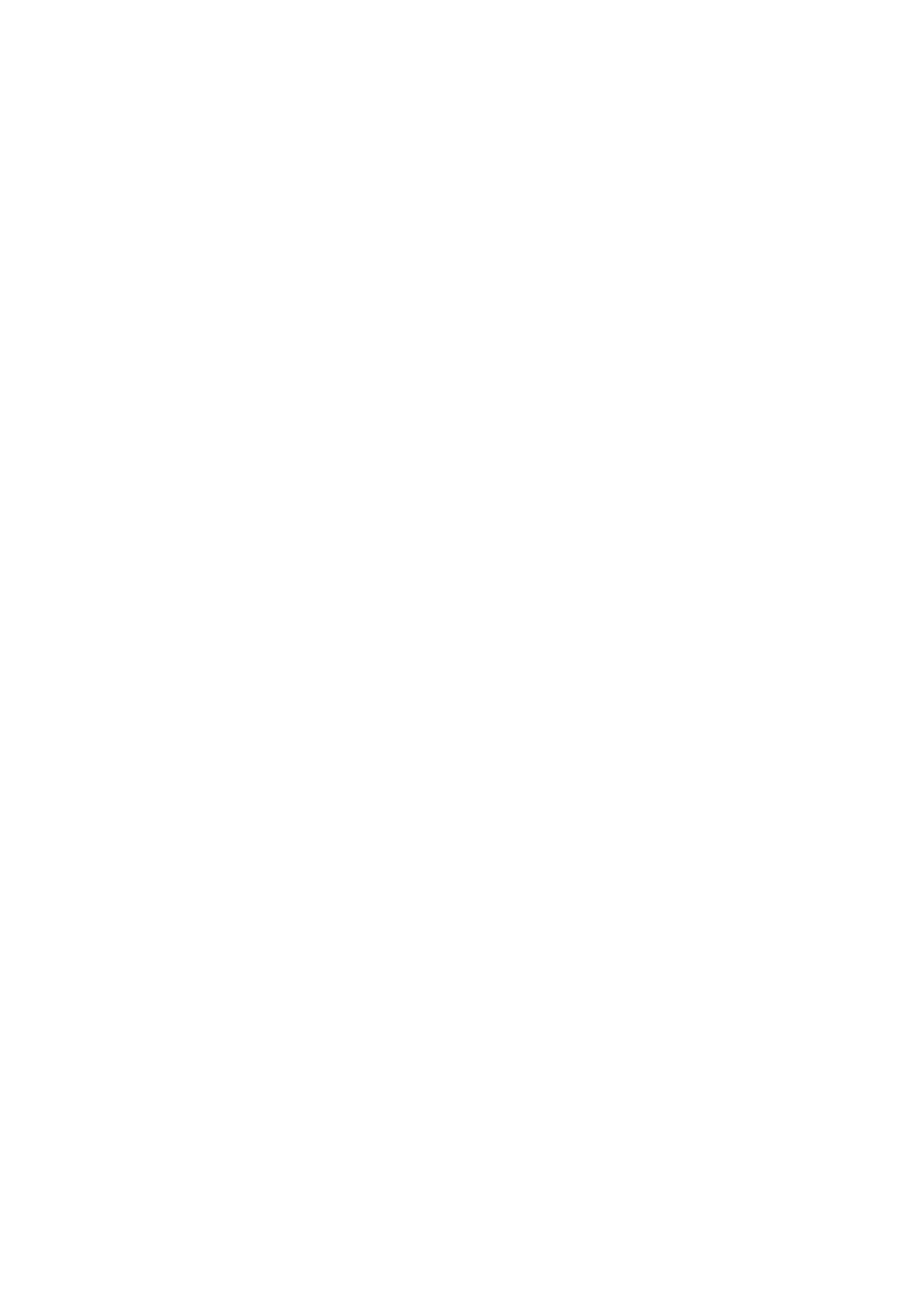

## Slide 5
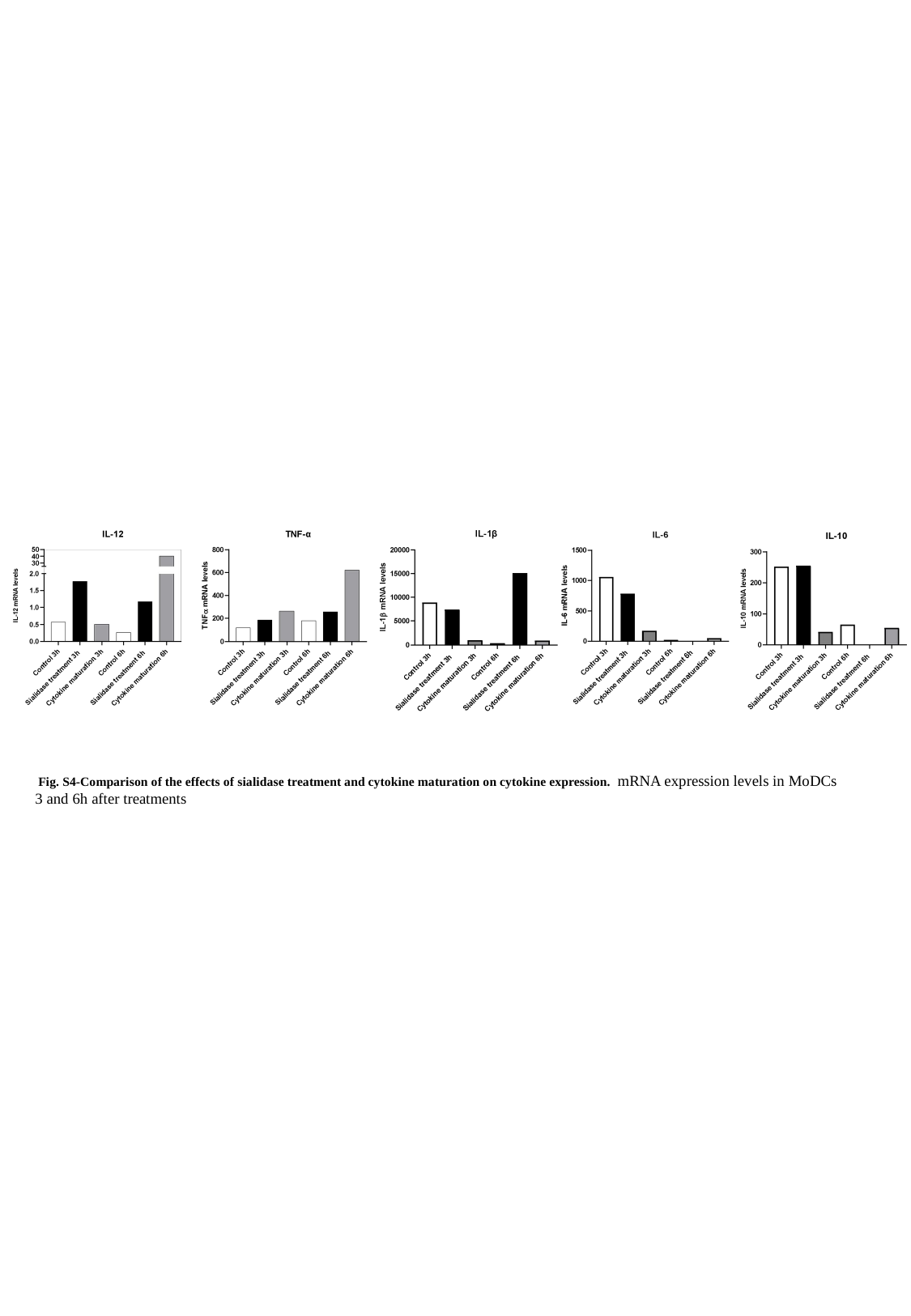

Fig. S4-Comparison of the effects of sialidase treatment and cytokine maturation on cytokine expression.  mRNA expression levels in MoDCs 3 and 6h after treatments
